# Can the use of azithromycin during labour reduce the incidence of infection among puerperae and newborns? A systematic review and meta-analysis of randomized controlled trials

**DOI:** 10.1186/s12884-024-06390-6

**Published:** 2024-03-14

**Authors:** Haiyan Ye, Jinlu Hu, Bo Li, Xia Yu, Xuemei Zheng

**Affiliations:** 1 Department of adult intensive care unite, School of Medicine, Chengdu Women’s and Children’s Central Hospital, University of Electronic Science and Technology of China, Chengdu, 611731 China; 2 Department of laboratory, School of Medicine, Chengdu Women’s and Children’s Central Hospital, University of Electronic Science and Technology of China, Chengdu, 611731 China

**Keywords:** Azithromycin, Labour, Sepsis, Infection, Puerperae, Newborns

## Abstract

**Objective:**

This systematic review and meta-analysis investigated whether the use of azithromycin during labour or caesarean section reduces the incidence of sepsis and infection among mothers and newborns.

**Data sources:**

We independently searched the PubMed, Web of Science, Cochrane Library and EMBASE databases for relevant studies published before February, 2024.

**Methods:**

We included RCTs that evaluated the effect of prenatal oral or intravenous azithromycin or placebo on intrapartum or postpartum infection incidence. We included studies evaluating women who had vaginal births as well as caesarean sections. Studies reporting maternal and neonatal infections were included in the current analysis. Review Manager 5.4 was used to analyse 6 randomized clinical trials involving 44,448 mothers and 44,820 newborns. The risk of bias of each included study was assessed using the criteria outlined in the Cochrane Handbook for Systematic Reviews of Interventions.Primary outcomes included the incidence of maternal sepsis and all-cause mortality and neonatal sepsis and all-cause mortality; secondary outcomes included maternal (endometritis, wound and surgical site infections, chorioamnionitis, and urinary tract infections) and neonatal outcomes (infections of the eyes, ears and skin). A random-effects model was used to test for overall effects and heterogeneity.

**Results:**

The pooled odds ratios (ORs) were as follows: 0.65 for maternal sepsis (95% CI, 0.55–0.77; *I*^*2*^, 0%; *P* < .00001); 0.62 for endometritis (95% CI, 0.52–0.74; *I*^2^, 2%; *P* < .00001); and 0.43 for maternal wound or surgical site infection (95% CI, 0.24–0.78; *P* < .005); however, there was great heterogeneity among the studies (*I*^2^, 75%). The pooled OR for pyelonephritis and urinary tract infections was 0.3 (95% CI, 0.17–0.52; *I*^2^, 0%; *P* < .0001), and that for neonatal skin infections was 0.48 (95% CI, 0.35–0.65; *I*^2^, 0%, *P* < .00001). There was no significant difference in maternal all-cause mortality or incidence of chorioamnionitis between the two groups. No significant differences were observed in the incidence of neonatal sepsis or suspected sepsis, all-cause mortality, or infections of the eyes or ears.

**Conclusion:**

In this meta-analysis, azithromycin use during labour reduced the incidence of maternal sepsis, endometritis, incisional infections and urinary tract infections but did not reduce the incidence of neonatal-associated infections, except for neonatal skin infections. These findings indicate that azithromycin may be potentially beneficial for maternal postpartum infections, but its effect on neonatal prognosis remains unclear. Azithromycin should be used antenatally only if the clinical indication is clear and the potential benefits outweigh the harms.

**Supplementary Information:**

The online version contains supplementary material available at 10.1186/s12884-024-06390-6.

## Introduction

Maternal infections, especially sepsis, account for 10% of maternal deaths during the perinatal period and are among the three major causes of maternal death worldwide [1].

It is estimated that 75,000 maternal deaths from infections occur worldwide each year, with the majority occurring in low-income countries [2]. Although the incidence reported by high-income countries is relatively low (0.1 to 0.6 per 1,000 births), it remains an important direct cause of maternal mortality [1]. Neonatal sepsis is the third most common cause of neonatal death, accounting for 16% of neonatal deaths, and maternal infection increases the risk of neonatal sepsis [1, 3]. An estimated 1 million neonatal deaths per year are related to maternal infections, with the majority of neonatal deaths occurring in low- and middle-income countries and regions [4, 5] and are mainly attributed to vertical transmission from the mother [6]. Therefore, perinatal prophylactic antibiotic use is essential to prevent postpartum maternal and neonatal infections and deaths.

Azithromycin is a macrolide antibiotic with a broad antimicrobial spectrum and good tissue penetration, which can effectively prevent and treat bacterial infections [7]. A single oral dose of azithromycin has been shown to significantly lower the risk of infections among expectant mothers and babies following vaginal delivery in various nations south of the Sahara [8]. Previous research has demonstrated that azithromycin, whether administered before caesarean section or vaginal birth, can lower the risk of maternal and newborn infections such as endometritis (RR = 0.38,95%CI = 0.34–0.42), wound infections (RR = 0.40,95%CI = 0.35–0.46), infant skin infections.

(RR = 0.49,95%CI = 0.25–0.93) and other serious complications (RR = 0.31,95%CI = 0.2–0.49), such as bacteraemia, sepsis, and death [8–10]. It can also reduce medical expenses [11]. However, some other studies have shown that azithromycin is not effective in reducing maternal and neonatal sepsis morbidity and mortality [8, 12–14]. Most of these studies had been conducted in resource-poor countries. There are conflicting findings on whether perinatal prophylaxis with azithromycin reduces maternal and neonatal infection and mortality. Azithromycin is an inexpensive, broad-spectrum antibiotic that is safe for use in mothers and newborns, does not require special storage conditions, and is not widely used in Africa [15]. Therefore, in terms of pharmacoeconomics, pharmacodynamics and ease of storage, we found it highly desirable to conduct a comprehensive meta-analysis to assess the impact of perinatal prophylaxis with azithromycin on maternal and neonatal outcomes.

## Data and methods

### Objective

The aim of this systematic review and meta-analysis was to evaluate the clinical significance and available evidence for oral or intravenous single-dose azithromycin during labour for the prevention of infections and deaths after vaginal delivery and caesarean section. Our systematic review and meta-analysis are registered with Prospero under registration number CRD42023442923. The Preferred Reporting Items for Systematic Reviews and Meta-Analyses (PRISMA) guidelines [16] were used to perform and report this systematic review and meta-analysis.

### Data sources and search strategy

Two authors independently searched for and reviewed eligible studies, assessed their risk of bias and extracted the data. Any queries were resolved through discussion by the review authors. We independently searched the PubMed, Web of Science, Cochrane Library and EMBASE databases for relevant studies published before February, 2024.The detailed search strategy is provided in the appendix (Supplementary Table 1). There were no language restrictions. We used the terms “azithromycin”, “intrapartum”, “infection”, “sepsis”, “maternal” and “newborn” to identify all randomized clinical trials (RCTs) that met the inclusion criteria.

### Eligibility criteria

We included RCTs assessing the effect of prenatal oral or intravenous azithromycin or placebo on infection rates and mortality after vaginal delivery or caesarean section. We included studies evaluating women who had vaginal births as well as caesarean sections. There was no special restriction on age, which was women of childbearing age. Studies reporting maternal and neonatal infections were included in the current analysis. We excluded studies in which both study groups received azithromycin, studies not reporting the rates of maternal or neonatal infections, nonrandomized studies, or studies with duplicate patient populations.

### Data extraction

Two independent reviewers screened the titles and abstracts of the identified studies for eligibility. Full-text searches were also conducted for potentially relevant studies. Any inconsistencies were resolved by discussion or negotiation between the two reviewers. We used our self-completed form to collect information from the literature, including the authors, year of publication, total number of study participants, and sample size of the intervention and control groups, weeks of pregnancy, drug dosage and outcome indicators, etc.

### Outcome measures

Primary outcomes included the incidence of maternal sepsis and all-cause mortality and neonatal sepsis and all-cause mortality; secondary outcomes included maternal (endometritis, wound and surgical site infections, chorioamnionitis, and urinary tract infections) and neonatal outcomes (infections of the eyes, ears and skin).

### Assessment of risk of bias

The risk of bias of each included study was assessed using the criteria outlined in the Cochrane Handbook for Systematic Reviews of Interventions [17]. Seven areas associated with risk of bias in each included trial were assessed because of evidence of problems associated with biased estimates of treatment effects: (1) randomized sequence generation; (2) allocation concealment; (3) blinding of participants and personnel; (4) blinding of outcome assessment; (5) incomplete outcome data; (6) selective reporting; and (7) other biases. The review authors judged the studies as “low risk”, “high risk” or “unclear risk” of bias.

### Statistical analysis

The Mantel‒Haenszel method was used to collect data using the random effects model [18]. The statistical heterogeneity between trials was evaluated by I^2^ statistics [18]. I^2^ statistics < 25%, 25–50% and > 50% were considered to indicate low, medium and high heterogeneity, respectively [18] Odds ratios (ORs) are used to describe the outcome indicators for categorical variables. Sensitivity analysis was performed after studies with an unclear or high risk of bias were excluded. The p value was two-tailed; *p* ≤ .05 was considered to indicate statistical significance. RevMan 5.4 software was used for statistical analysis.

## Results

**Fig. 1:**
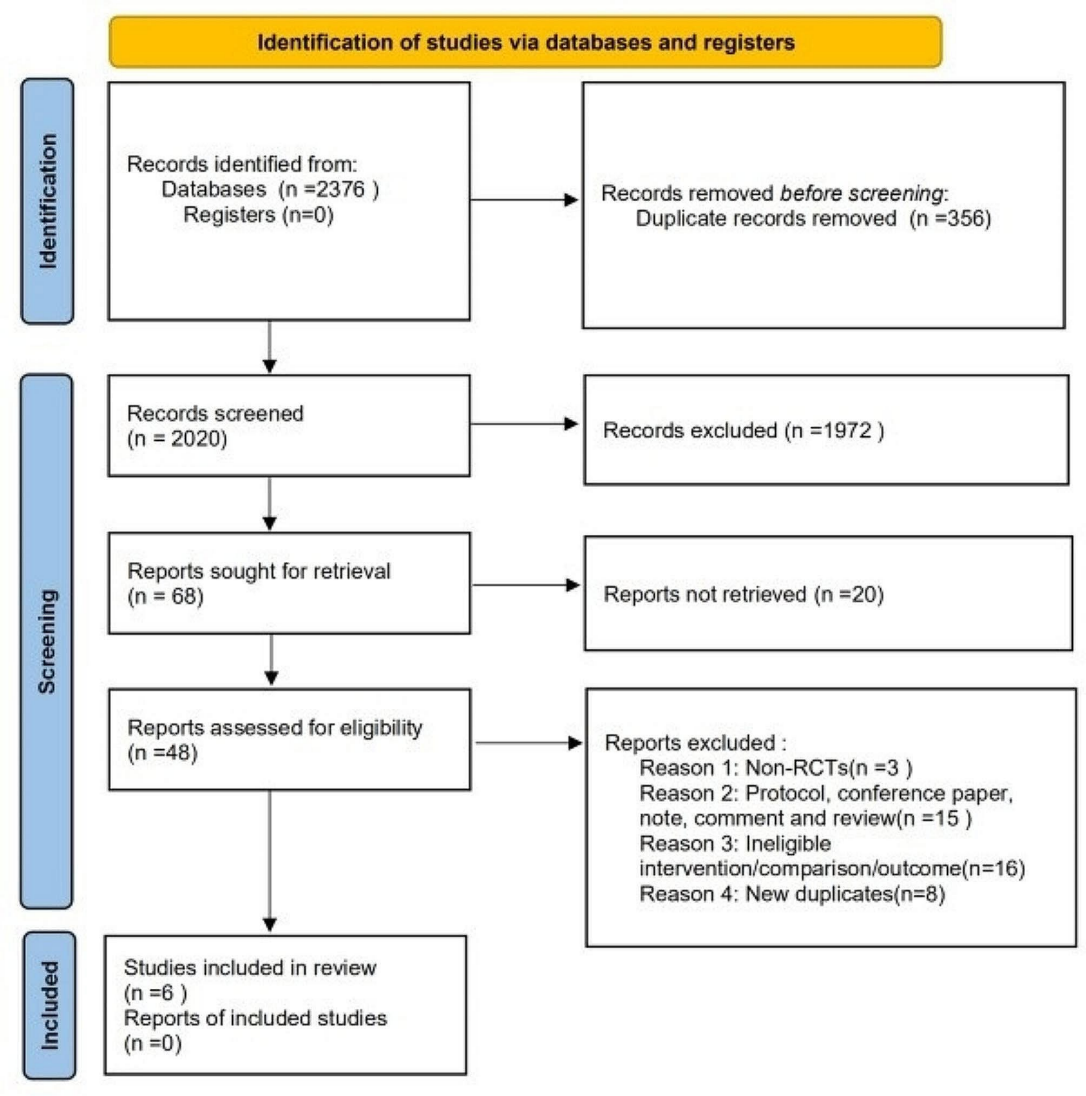
Flow diagram of studies identified in the systematic review

The study selection process is shown in Fig. 1. A total of 44,448 mothers and 44,820 newborns from 6 RCTs were included in the final analysis [8, 13, 14, 19–21].

### Study characteristics

Six studies, four of which were multicentre studies were RCTs [13, 14, 20, 21]. The geographical distribution of the study population was extensive.One study was performed in the United States [13]. The other studies were carried out in low- and middle-income regions in Latin America, Asia, and Africa, including Cameroon, India, Gambia, and other regions [8, 14, 19–21]. In two studies [13, 19], the experimental group received a single intravenous injection of 500 mg of azithromycin in 250 ml of saline, whereas the control group received a single intravenous injection of normal saline with the same appearance. Jyothi et al. [19] additionally injected a single dose of 2 g cefazolin intravenously into the experimental group and the control group. The intervention measures in the other four trials were a single oral dose of azithromycin, either 1 g [14] or 2 g [8, 20, 21]. The basic characteristics of the included studies are shown in Tables 1 and 2.

**Table 1:** Baseline characteristics of include trials

Author	Single vs. Multicenter	Regions	Average age(Years) (I/C)	Gestational age(Weeks)(I/C)	Duration of rupture of membranes (Hours) (I/C)	Sample size(n)	
						Azithromycin group (M/N)	Placebo group (M/N)
Tita 2016	Multicenter	America	28.2 ± 6.1/ 28.4 ± 6.5	38.9 ± 2.3/ 39.0 ± 2.3	NA	1,019/ 1,019	994/994
Oluwalan-a 2017	Single	Gambia	IQR: 26(22–30)/ 25(22–30)	IQR: 36(35–38)/ 36(35–38)	0.4(0.1–1.8)/ 0.3(0.1–1.3)	414/419	415/424
Jyothi 2019	Single	Chandigarh and India	26.42 ± 2.65/ 27.39 ± 3.03	36.41 ± 2.77/36.75 ± 2.71	4.48 ± 3.87/ 4.34 ± 4.01	100/100	100/100
Subrama-niam 2021	Multicenter	Cameroon	27.2 ± 5.3/ 26.0 ± 5.3	39.4 ± 1.5/ 39.2 ± 1.4	15.7(2.6–29.7)/15.6 (3.4–27.7)	253/257	250/255
Roca 2023	Multicenter	Gambia and Burkina Faso	IQR: 27(22–31)/ 26 (22–31)	NA	0.3(0.1–1.5)/ 0.3 (0.1–1.4)	5,802/ 5,889	5,823/ 5,894
Tita 2023	Multicenter	Africa,Asia and Latin America	IQR: 24(21–28)/ 24 (21–28)	38.9 ± 2.3/ 39.0 ± 2.3	NA	14,590/ 14,687	14,688/ 14,782

I = intervention group;C = control group;M = mothers;N = newborns;NA = Not applicable;

IQR = interquartile range

**Table 2:** Baseline characteristics of include trials

Author	Route of Admin-istration	Dosing Regimen (I/C)	Follow-up (M/N)	Hours Between Treatment and Delivery (T/C)	Mode of Delivery
Tita 2016	Intrave-nous	A dose of 500 mg in 250 ml of saline/ An identical appearing saline	6 weeks after surgery/With 28 days or 3 months	Once the decision was made to proceed to cesarean section	Cesarean
Oluwalana 2017	Oral	A single dose of 2 g azithromycin/ A single dose of 2-g placebo	Postpartum 8 weeks	IQR: 3.2(1.1–8.3)/ 2.9 (1.3–6.3)	Vaginal or Cesarean
Jyothi 2019	Intrave-nous	A single dose of 2-g cefazolin and 500 mg azithromycin, prior to skin incision/ A single dose of 2-g cefazolin and placebo before the skin incision	Postoperative 6 weeks	NA	Cesarean
Subramaniam 2021	Oral	1-g azithromycin and placebo/ Placebo and placebo	At delivery hospitalization or up to 6 weeks after delivery	5.6(1.7–17.9)/ 6.5 (1.6–15.1)	Vaginal or Cesarean
Roca 2023	Oral	Azithromycin (2-g)/ placebo	Postpartum 28 days	1.6(0.5-4.0)/ 1.6 (0.5–4.3)	Vaginal or Cesarean
Tita 2023	Oral	A single dose of 2-g azithromycin/ Identical placebo	Within 6 weeks after delivery	NA	Vaginal or Cesarean

I = intervention group;C = control group;M = mothers;N = newborns;NA = Not applicable;

IQR = interquartile range

### Risk of bias of the included studies

**Fig. 2:**
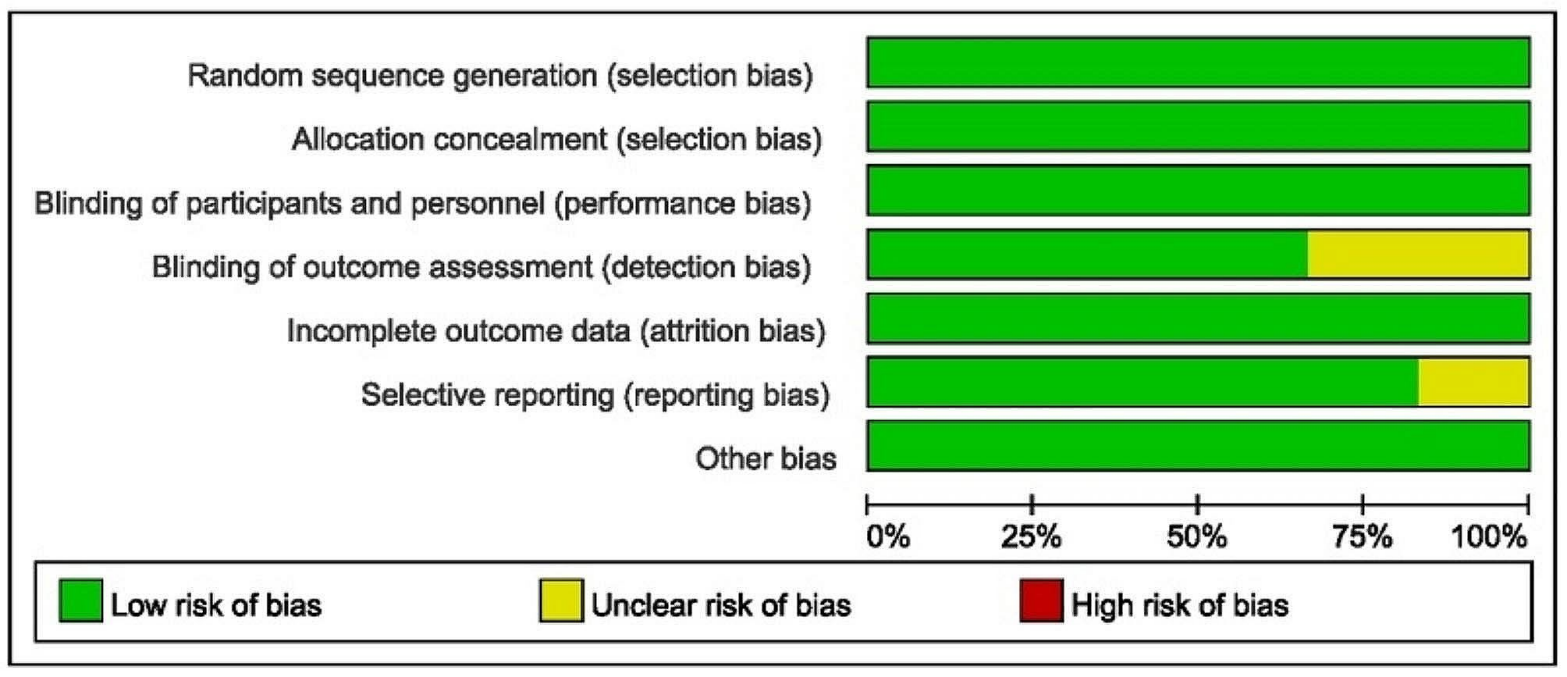
Risk of bias

All six trials were classified as having a low risk of bias (Fig. 2).

#### Primary outcomes

**Fig. 3:**
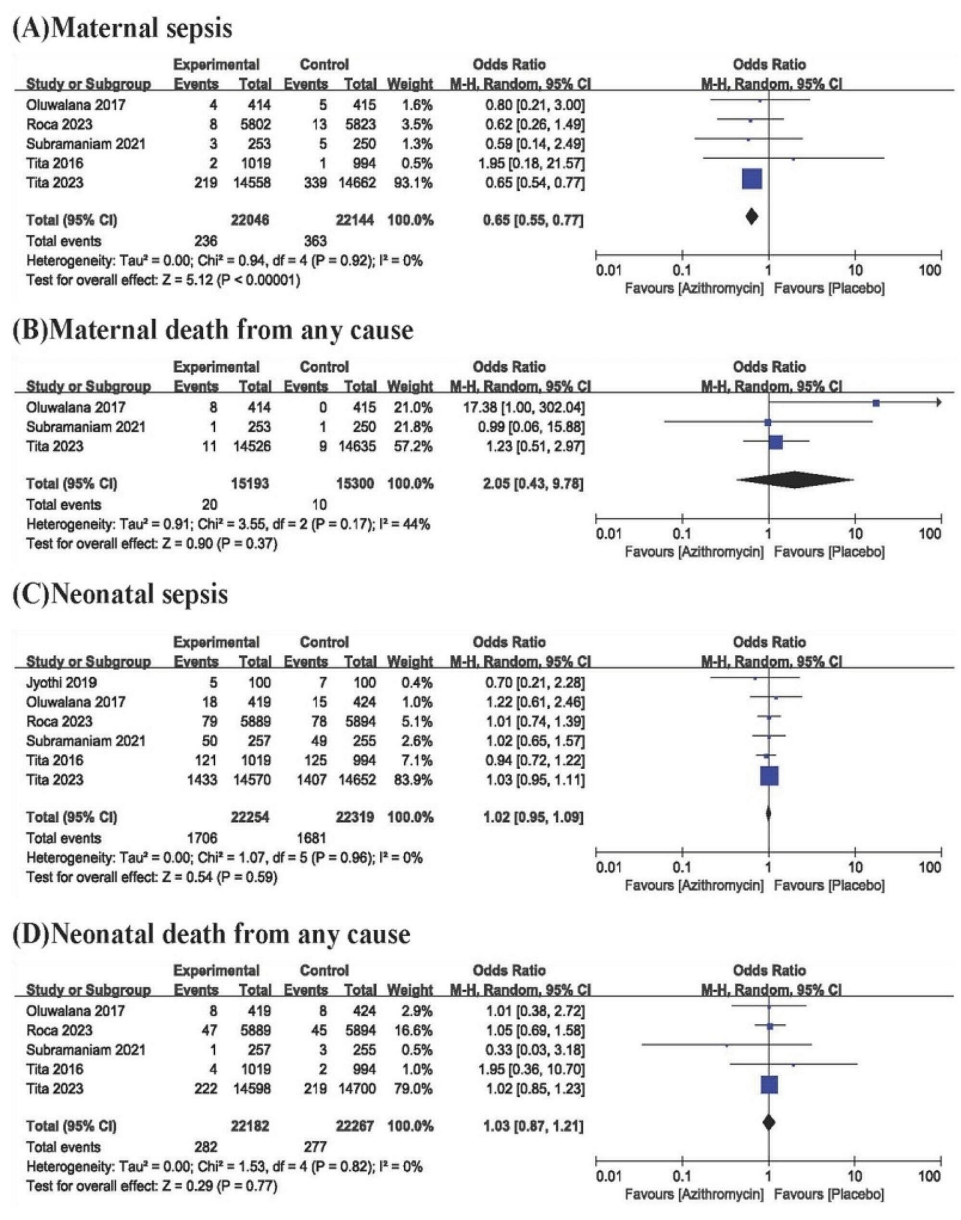
Forest plot for primary outcomes

Among the six studies, five analysed the incidence of maternal sepsis. In the studies of Tita et al. [21] and Subramaniam et al., maternal sepsis was defined according to the World Health Organization (WHO) criteria, while the other three studies did not explicitly mention it. The incidence of maternal sepsis was significantly lower in the azithromycin group than in the control group (OR, 0.65; 95% CI, 0.55–0.77; I^2^, 0%; *P* < .00001) (Fig. 3). Maternal death from any cause was analysed in three studies.

There was no statistically significant difference in maternal death from any cause between the azithromycin group and the control group (OR, 2.05; 95% CI, 0.43–9.78; I^2^, 44%; *P* < .37) (Fig. 3). All studies mentioned neonatal sepsis, and the diagnosis of neonatal sepsis in two studies [14, 21] was based on WHO criteria. There was no statistically significant difference in neonatal sepsis between the azithromycin group and the control group (OR, 1.02; 95% CI, 0.95–1.09; I^2^, 0%; *P*<.59) (Fig. 3). Neonatal death from any cause was analysed in five studies. There was no statistically significant difference in neonatal death from any cause between the azithromycin group and the control group (OR, 1.03; 95% CI, 0.87–1.21; I^2^, 0%; *P*<.77) (Fig. 3).

#### Secondary outcomes

**Fig. 4:**
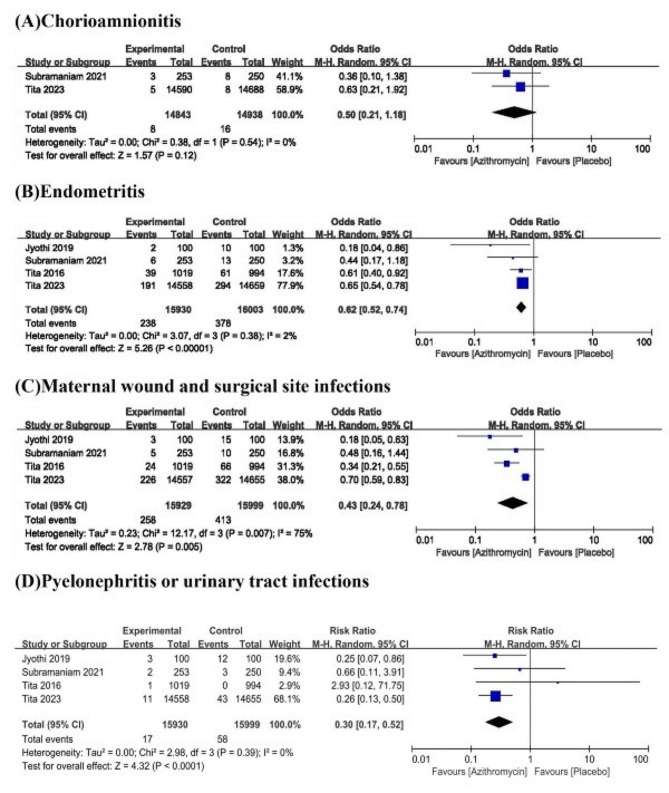
Forest plot of secondary outcomes for mothers

Maternal chorioamnionitis was analysed in 2 studies. There was no statistically significant difference in the incidence of maternal chorioamnionitis between the azithromycin group and the placebo group (OR, 0.5; 95% CI, 0.21–1.18; I^2^, 0%; *P* = .12) (Fig. 4). Indicators of maternal endometritis were described in four trials. Analysis revealed a lower risk of maternal endometritis in the azithromycin group than in the placebo group (OR, 0.62; 95% CI, 0.52–0.74; I^2^, 2%; *P* < .00001) (Fig. 4).

**Fig. 5:**
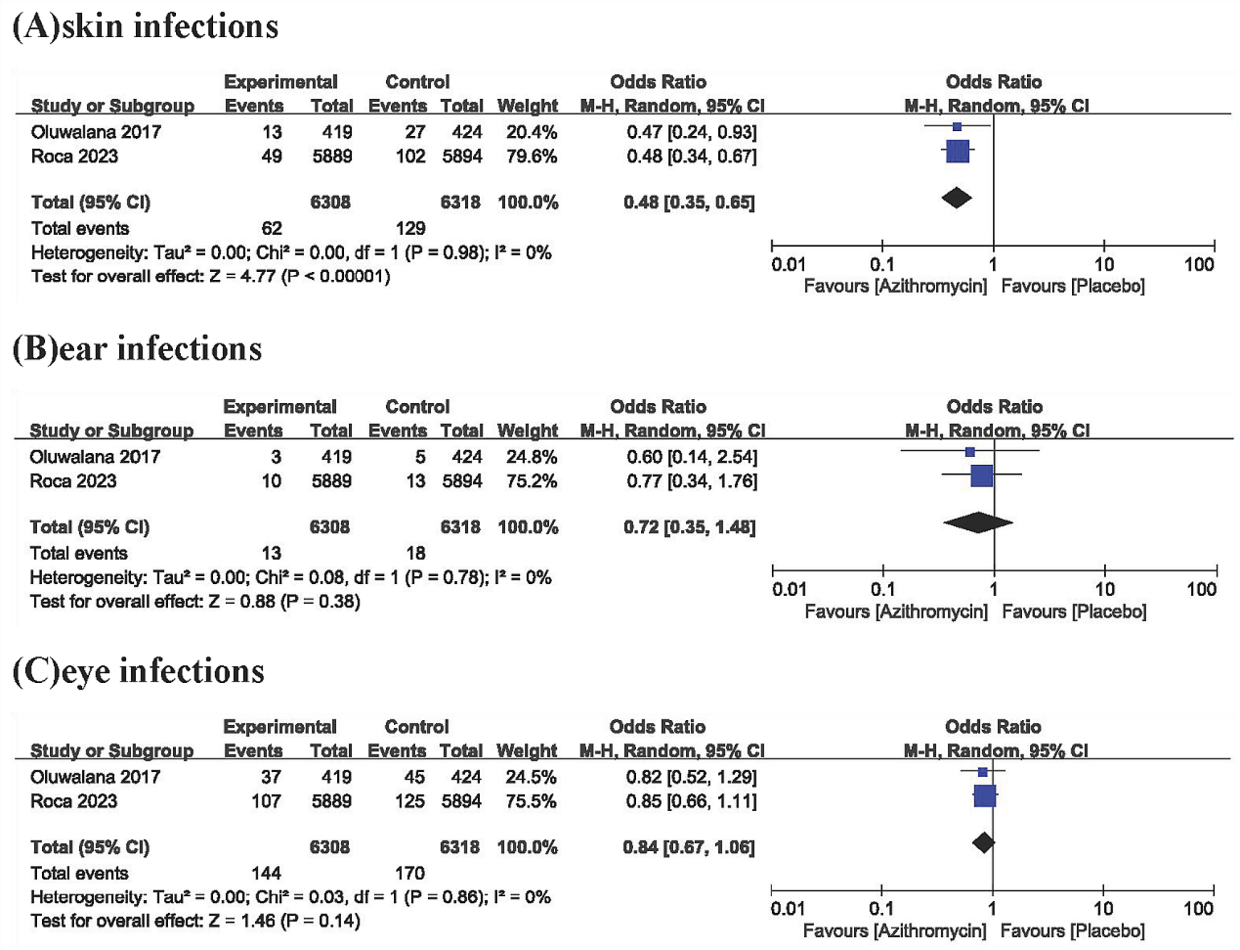
Forest plot of the secondary outcomes of the newborns

Maternal wound and surgical site infections were evaluated in 4 studies. The results showed that there were significant differences in maternal wound and surgical site infections between the azithromycin group and the placebo group (OR, 0.43; 95% CI, 0.24–0.78; *P* < .005) (Fig. 4), but there was great heterogeneity among the studies (I^2^, 75%) (Fig. 4). A step-by-step sensitivity analysis was performed to evaluate the studies leading to heterogeneity. After excluding the Azithromycin to Prevent Sepsis or Death in Women Planning a Vaginal Birth [21] study, the observed heterogeneity was resolved, and similar conclusions were drawn (OR, 0.33; 95% CI, 0.22–0.5; I^2^, 0%; *P* < .00001). Four studies that included outcome metrics for pyelonephritis or urinary tract infections showed statistically significant results in the azithromycin group (OR, 0.3; 95% CI, 0.17–0.52; I^2^, 2%, *P* < .0001) (Fig. 4). Other newborn outcome indicators were described in only the studies by Roca et al. and Oluwalana et al. Azithromycin reduced the incidence of neonatal skin infections compared with placebo (OR, 0.48; 95% CI, 0.35–0.65; I^2^, 0%, *P* < .00001) (Fig. 5), but no statistically significant differences were observed in neonatal ear or eye infections between the two groups (Fig. 5).

## Discussion

In this meta-analysis of 6 randomized clinical trials, including 44,448 mothers and 44,820 newborns, we compared the effects of a single oral or intravenous dose of azithromycin administered antenatally or in the intrapartum period versus placebo on the incidence of maternal and neonatal sepsis and all-cause mortality. The key findings of this analysis were as follows: (1) compared with placebo, azithromycin reduced the risk of postpartum sepsis among mothers but not the incidence of neonatal sepsis; (2) azithromycin was not effective at reducing all-cause mortality rates among both mothers and neonates; (3) azithromycin was associated with a lower risk of endometritis, wound and incision infections, and urinary tract infections, but there was no significant difference in the risk of chorioamnionitis compared with the placebo; and (4) azithromycin reduced the incidence of neonatal skin infections but was not associated with neonatal infections of the eyes or ears.

Our meta-analysis showed that receiving azithromycin during labour and delivery reduced the risk of maternal sepsis. This result comes mainly from a multinational RCT study by Tita et al. [21] Azithromycin reduced the risk of sepsis after caesarean section and vaginal delivery, with greater maternal benefit in Africa than in Asia. This discrepancy may arise from the fact that azithromycin is not widely used in Africa [15]. Two recent systematic evaluations [22, 23] have shown that the addition of a single dose of azithromycin at the time of delivery is associated with a reduced risk of maternal sepsis. One of them evaluated indicators related to the prophylactic oral administration of a single dose of azithromycin antenatally in women with planned vaginal deliveries. Our meta-analysis was consistent with the results of these studies and included similar studies, all of which included the study by Tita et al. [21] As a result, the statistical validity and reliability of the observations are improved due to our more diverse and wider sample size.

Our maternal results are consistent with the findings of several studies on azithromycin in women undergoing caesarean section and conventional medication [24–26]. These studies, a single-center randomized trial involving 597 women and follow-up observational studies from the same center, showed that women who received azithromycin-based broad-spectrum antibiotic prophylaxis after cord clamping had at least a 30% lower rate of postoperative infections than those who received standard prophylaxis, as well as a reduced incidence of endometritis and wound infections after caesarean section. This may be related to the fact that caesarean section itself can increase postpartum infections [27]. A meta-analysis of 95 randomized controlled trials [10] showed that the use of prophylactic antibiotics in women undergoing caesarean section reduced the incidence of wound infections, endometritis and serious infectious complications by 60–70%. Our findings are in line with these studies, but we had high heterogeneity among the studies evaluating wound infections. This difference may stem from the different routes of delivery. The observed heterogeneity was resolved after the exclusion of the studies of azithromycin to prevent sepsis or death in women planning a vaginal birth, which are considered to be related to the fact that the characteristics of the population in this study were mainly transvaginal births, whereas the other studies were mostly cesarean deliveries. There are few studies on the use of azithromycin for the prevention and treatment of maternal urinary tract infections, but our study shows that azithromycin appears to reduce the risk of urinary tract infections, especially pyelonephritis, which may require larger randomized controlled trials to confirm the findings. A study that included 1289 women treated with erythromycin and azithromycin for preterm premature rupture of membranes [28] showed a lower risk of chorioamnionitis in the azithromycin group, which is inconsistent with the results of our study. The reason may be that chorioamnionitis was reported in only two studies and the number of events occurring was very low, so the result needs to be interpreted with caution.

Our meta-analysis showed that neonatal mortality and sepsis rates did not benefit from maternal use of azithromycin during labour. This is similar to the study by Oldenburg et al. [29] In this RCT, neonates aged 8 to 27 days were randomly assigned to either the placebo or azithromycin group. Finally, there was no difference in the 6-month or 12-month infant mortality rates between the two groups. Another study [30] showed no evidence of early-onset sepsis in term newborns who had not been exposed to antibiotics in the perinatal period, while at least one infant was diagnosed with early-onset sepsis in term or preterm newborns who had been exposed to antibiotics before birth. This may be due to the fact that intrapartum azithromycin is unlikely to be effective against infection in the first few hours of neonatal life, and that participants in the trials had a short time interval between azithromycin administration and delivery (median 1.6 h) [20]. Our findings did not show a benefit on neonatal sepsis or mortality, but it significantly reduced skin infections in neonates. Azithromycin is an effective and tolerable alternative to first-line drugs for the treatment of skin in children [31]. A study by ROCA et al. [20] showed a reduction of more than 50% in neonatal skin infections, including those requiring hospitalisation, which may reflect the impact of the intervention on S. aureus and Streptococcus colonisation in neonates [12].

Increased risk of major congenital malformations, spontaneous abortions, gastrointestinal malformations, cardiovascular malformations, preterm delivery, and low birth weight have been reported in several studies [32–37] of fetal and neonatal outcomes following prenatal azithromycin exposure. However, There is no established evidence to support the use of azithromycin in pregnant women with adverse consequences for their offspring [38]. Therefore, although azithromycin may have potential benefits for maternal health, its impact on neonatal outcomes remains unclear. If the expected therapeutic benefit outweighs the potential risk, the drug should be used during pregnancy only if clinically indicated [38].

Certain caveats should be considered when interpreting the results of the current meta-analysis. First, the results of this meta-analysis were largely dominated by one study (i.e., the Tita2023 trial), which may have resulted in a heavy reliance on the trial for some outcome metrics and may have led to inaccurate results in the systematic evaluation. Second, there was significant heterogeneity in some of the research results, as the studies differed in sample size and inclusion criteria; therefore, no definitive conclusions could be drawn from the available randomized data, and additional studies are needed to further elucidate the value of antenatal azithromycin use compared with placebo for maternal and neonatal infections. Third, in this meta-analysis, azithromycin was administered both orally and intravenously, and the dose of azithromycin was not consistent, which may lead to inaccuracies in the results of the systematic evaluation due to the small number of included studies, which did not allow subgroup analyses or meta-regressions to be performed on a step-by-step basis. Fourth, out of the six RCTs included in the meta-analysis, only one was conducted in the U.S., while the remaining five were conducted in low-income regions such as Africa and Latin America. Since the economic and medical conditions in low-income countries such as Africa and Latin America vary greatly, this meta-analysis may only reflect the treatment situation in these regions, and additional RCTs are needed to validate these results in developed countries such as the United States. Fifth, we did not evaluate the outcome indicator of overall maternal and neonatal infections because some of the included studies did not explicitly provide data on overall maternal and neonatal infections. These data could be further assessed in future studies.

## Conclusions

In this meta-analysis, azithromycin use during labour reduced the incidence of maternal sepsis, endometritis, incisional infections and urinary tract infections but did not reduce the incidence of neonatal-associated infections, except for neonatal skin infections. These findings indicate that azithromycin may be potentially beneficial for maternal postpartum infections, but its effect on neonatal prognosis remains unclear. Azithromycin should be used antenatally only if the clinical indication is clear and the potential benefits outweigh the harms.

### Electronic supplementary material

Below is the link to the electronic supplementary material.

Supplementary Material 1

Supplementary Material 2

## Data Availability

No datasets were generated or analysed during the current study.

## Abbreviations

WHO: World Health Organization
RCTs: Randomized clinical trials
